# Assessment of *ab initio* models of protein complexes by molecular dynamics

**DOI:** 10.1371/journal.pcbi.1006182

**Published:** 2018-06-04

**Authors:** Filip Radom, Andreas Plückthun, Emanuele Paci

**Affiliations:** 1 Department of Biochemistry, University of Zurich, Zurich, Switzerland; 2 Astbury Centre for Structural Molecular Biology, University of Leeds, Leeds, United Kingdom; University of Maryland School of Pharmacy, UNITED STATES

## Abstract

Determining how proteins interact to form stable complexes is of crucial importance, for example in the development of novel therapeutics. Computational methods to determine the thermodynamically stable conformation of complexes from the structure of the binding partners, such as RosettaDock, might potentially emerge to become a promising alternative to traditional structure determination methods. However, while models virtually identical to the correct experimental structure can in some cases be generated, the main difficulty remains to discriminate correct or approximately correct models from decoys. This is due to the ruggedness of the free-energy landscape, the approximations intrinsic in the scoring functions, and the intrinsic flexibility of proteins. Here we show that molecular dynamics simulations performed starting from a number top-scoring models can not only discriminate decoys and identify the correct structure, but may also provide information on an initial map of the free energy landscape that elucidates the binding mechanism.

## Author summary

Determining how proteins fold and form complexes is of crucial importance, for example in the development of novel therapeutics. Experimental determination of structures is costly and lengthy. Computational methods to determine the thermodynamically stable conformation of complexes from the structure of the binding partners are available and constantly improving. Such methods generate a large number of diverse conformations and rank them for their likelihood to be correct. Even a model very similar to the correct structure is rarely the top-scoring one, but, as in the examples presented here, only within the top ~10–100 (the exact number depends on the complexity of the structure, and could be much higher). Here we show through atomistic simulation that good models are kinetically stable and bad models most often are not. More surprisingly, we also see that some bad models spontaneously find the correct (i.e., experimentally determined) conformation. This is remarkable, and could become an additional tool to contribute to structure determination of protein complexes. Such a result can also be expected, because evolution sculpted the free energy landscape in a way that the biologically active state is not only the one of lowest free energy (i.e., the most likely state) but also robustly reachable and kinetically stable (i.e., at the bottom of a funnel on the free energy landscape).

## Introduction

Most biological processes are mediated by interactions between proteins. The high-resolution structure of protein complexes may help understand those processes at the molecular level and possibly interfere with them by rational design.

Predicting structures of complexes from known protein structures is, at first sight, a simpler task than predicting protein structures from sequences, i.e., *ab initio*. Yet, the facts that proteins are intrinsically flexible, and that they may change conformational propensity when interacting with other proteins, complicate the task. This entails changes from the very small to the very large (rotamers of interacting amino acids, movements of loops, up to domain orientations, and all combinations of them). Nevertheless, *ab initio* structure prediction methods constantly improve and can at present, at least for some relatively small systems, generate models that are indistinguishable from the experimental structure of the complex. Unfortunately, this is not the rule, however.

A number of different algorithms have been developed to dock proteins, including ZDOCK[1], PatchDOCK[2], ClusPro[3], ATTRACT[4], Gramm-X[5], DOCK/PIERR[6] and RosettaDock[7]. Some of them, including HADDOCK[8] or CamDock[9], are guided by experimental data. Their performance is assessed periodically in the Critical Assessment of Predicted Interactions (CAPRI), where different research groups compete in blind docking of diverse complexes[10].

Biochemical[11, 12] or evolutionary data[13, 14] may provide the constraints to navigate docking or evaluate decoys. In their absence one usually ends up with dozens of model candidates with similarly good scores. One reason is that scoring functions are only rough approximations of the free energy. Another reason is that many structures exist that are energetically close but structurally very different. Hence, the great challenge is to identify the correct model, i.e., the near-native structure(s).

For this purpose, several rescoring/re-ranking algorithms with different energy functions were developed. One of them is ZRANK which improves the ranking of the near-native poses across a benchmark set of different complexes[15]. FiberDock[16] or GalaxyRefine[17] perform additional backbone and side-chain relaxations. Different metrics to score and re-rank the docked poses are reviewed in Ref. [18]. Although rescoring often helps to narrow down the pool of model candidates, it usually does not unambiguously direct to the correct structure.

Molecular dynamics (MD) simulations have been widely used to study the dynamics of proteins on time scales that are, almost always, orders of magnitude shorter than the folding and binding times. On such short timescales MD may be used to assess the stability of structures of complexes obtained from small molecule docking[19]. In protein-protein docking, MD served typically for the local refinement of near-native decoys[17, 20]. Combined with Markov modeling, MD has been shown to be a powerful tool to recapitulate association kinetics[21]. A crucial property of a correctly docked conformation is that it would be expected to be near the bottom of a funnel in the free energy landscape, separated by sizeable barriers in free energy from incorrect conformations. Wrongly docked conformations, instead, may be either unstable or metastable conformations.

Here, we show that atomistic simulations, starting from a number of diverse, high-scoring models, provide valuable information on the local properties of the free-energy landscape that can be used to discriminate near-native from non-native protein-protein docking poses. For two different complexes, the Designed Ankyrin Repeat Protein G3 (DARPin G3) bound to domain IV of Human Epidermal Growth Factor Receptor 2 (HER2_IV)[22] and extracellular fibrinogen-binding protein (Efb-C) bound to C3–inhibitory domain of *Staphylococcus aureus* (C3d)[23], we show that the majority of decoys with reasonable scores in the initial docking are kinetically unstable and diffuse away from their initial conformation.

Remarkably, some decoys happen to be within the binding funnel on the free energy landscape and diffuse in our simulations to the correct structure on sub-μs timescales. Thus, these methods appear to capture binding events for models way off the correct structure that may even be trapped intermediates within the binding event. Such binding trajectories may thus also provide valuable information on the free energy landscape and the binding mechanism.

## Results

### DARPin G3:HER2_IV complex

We used RosettaDock in its simplest form, without constraints nor post-processing, to generate a number of poses to be used as starting conformations for molecular dynamics simulations.

All-atom, fully solvated, molecular dynamics simulations were started from each of the best 50 models produced by RosettaDock and have been performed with the assumption that decoys are unstable or metastable states, and the trajectory will thus drift away from the initial structure.

**Fig 1:**
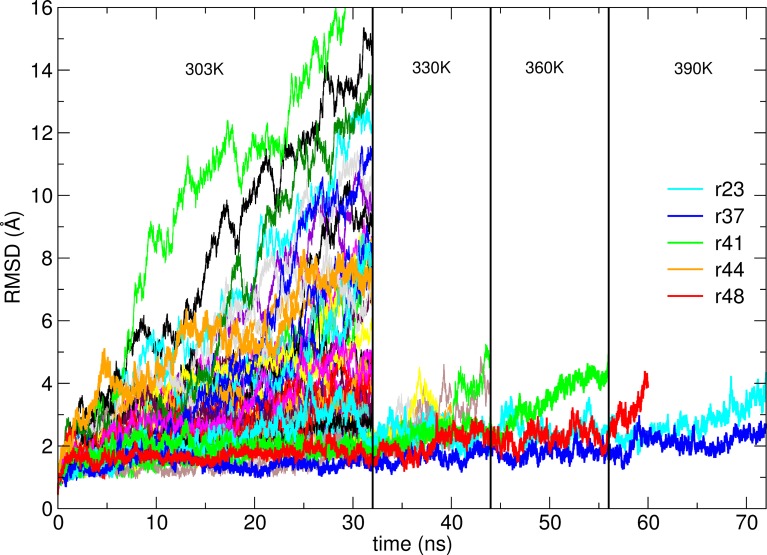
Root-mean-square deviation from the position of Cα atoms in the respective models of G3:HER2_IV complex as a function of simulation time. While some models rapidly move away from the initial pose, others only deviate from the initial structure when temperature is increased. The only model that even after 20 ns simulation at a temperature of 390 K remains close to the initial structure is model r37, that is, the one closest to the correct structure.

During the room temperature simulations, we observed that most of the high scoring decoys indeed drift away from the initial configuration. After 32 ns at 303 K, 38 out of 50 models are more than 2.5 Å away from their initial structure (Fig 1).

**Fig 2:**
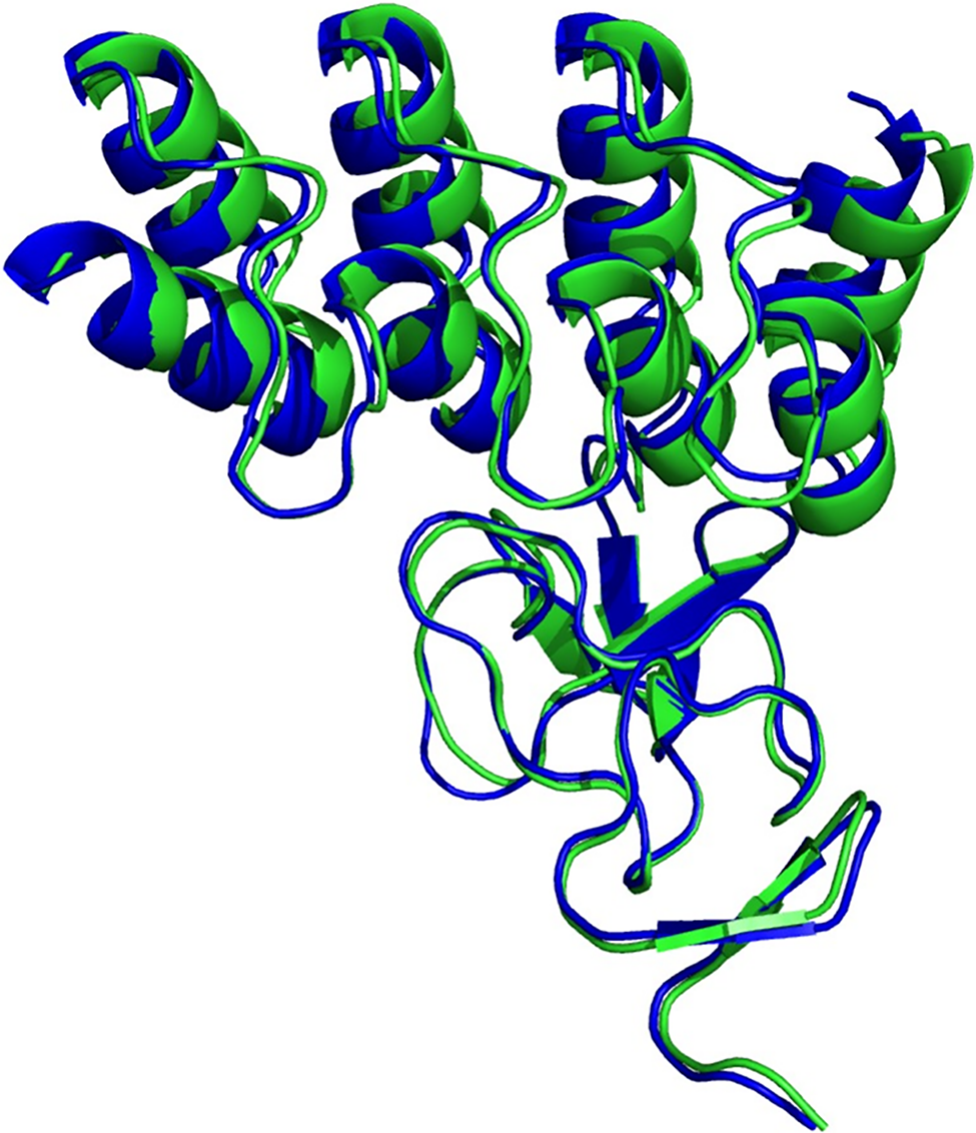
Model r37 (blue) aligned to crystal structure of the G3:HER2_IV complex (green). The RMSD between the two is 0.8 and 1.7 Å if the backbone atoms or all the atoms are considered, respectively.

To challenge the structures that during the simulation may get trapped into metastable conformations we increased the temperature by 30 K intervals and continued the simulations for 12 ns for each temperature. After 20 ns at 390 K, only model r37 remains within 2.5 Å RMSD from the initial structure. Model r37 is effectively the model by far the closest to the experimental structure (Fig 2). Ligands in other models cover the vast surface space of the receptor, including regions completely unrelated to the epitope (S1 Fig).

Interestingly, models r41, r48 and especially r23 appear to be kinetically stable, even when simulations are continued at higher temperatures, and only start deviating from the initial conformation when the temperature is raised to 390 K. The kinetic stability of r23 appears to be maintained mainly by Y46 of the DARPin fitting into the proline-rich hydrophobic pocket (P529, P543, P547) of the target, supported by the spatially neighboring R23 that may bridge with E544 (Supplementary Information S2 Fig).

**Fig 3:**
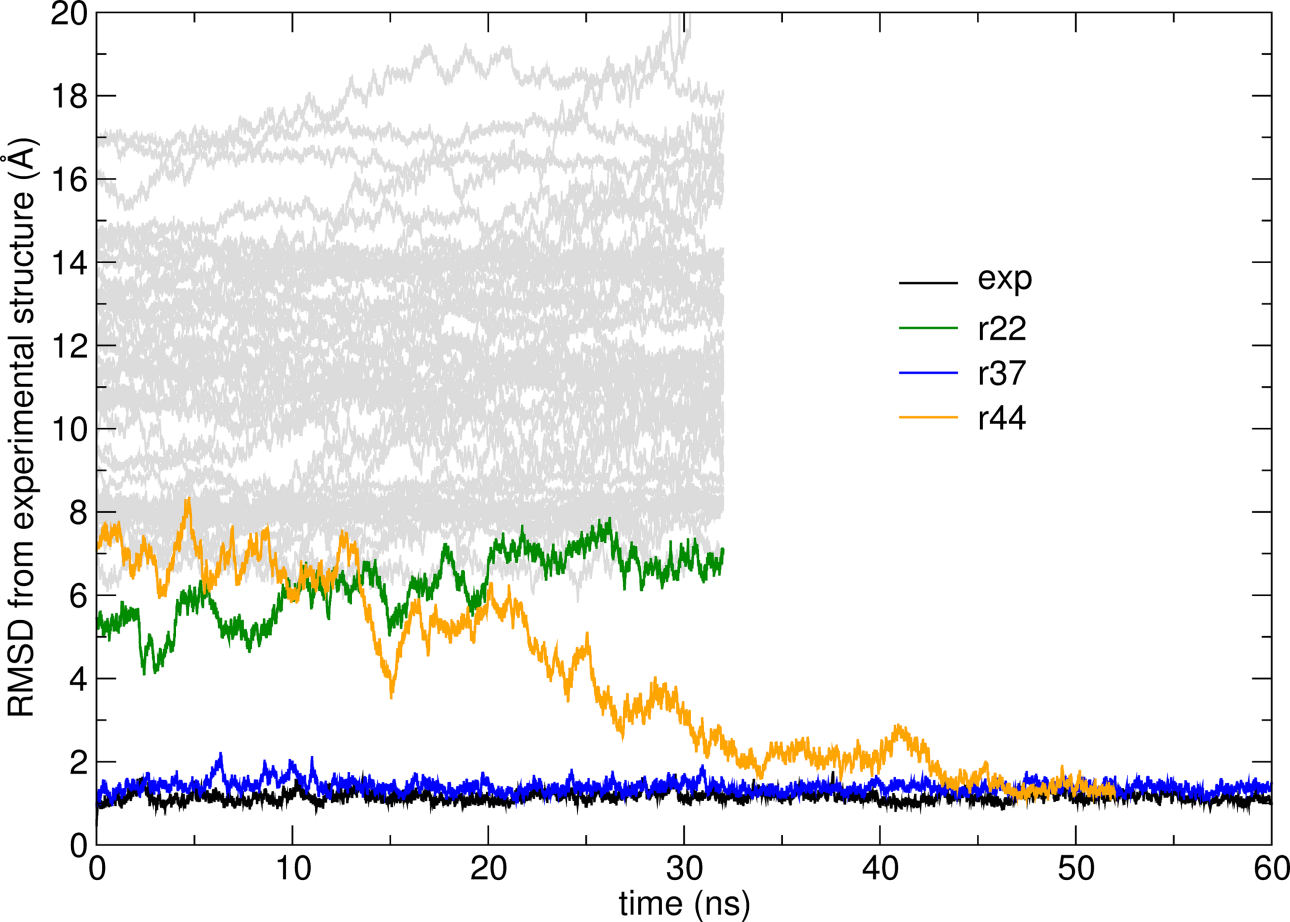
RMSD (for Cα atoms) from the reference experimental structure as a function of time along simulations at 303 K for the G3:HER2_IV complex. Simulations starting from model r37 or from the experimental structure explore a narrow range of conformations close to the experimental structure. The simulation starting from model r44 converges to the correct structure after about 50 ns, suggesting that, despite the remarkable structural difference from the correct structure, model r44 is within the native basin of the free energy surface. All the other trajectories do not lead to the correct state after 32 ns simulation. A simulation started from the experimental structure is also shown: the RMSD fluctuates around 1 Å indicating high rigidity of the complex.

The root-mean-square deviation from the experimental structure for each of the simulations (at 303 K) starting from the different models is shown in Fig 3. The simulation starting from model r37, which was shown already to be very close to the experimental structure, converges to the correct bound state after the initial equilibration.

Interestingly, model r44, initially at about 8 Å RMSD from the experimental structure, starts moving towards the experimental structure after about 15 ns and becomes indistinguishable from it after about 50 ns. This suggests that r44, while considerably off-target, can slide into the correct conformation without encountering sizeable free energy barriers. In other words, the model is likely to fall within a broad funneled region on the free energy landscape that corresponds to the correct bound state.

**Fig 4:**
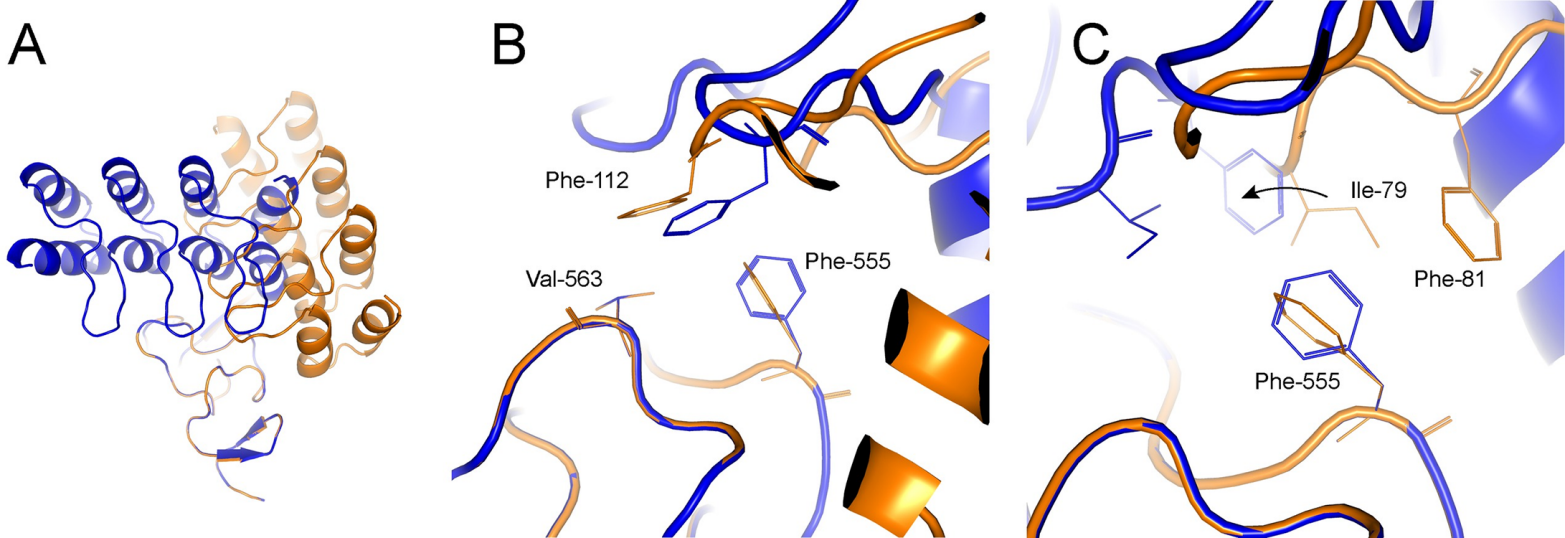
Model r37 (blue) and r44 (orange) of G3:HER2_IV show similarity at the C-terminal region. **(A)** Model overview **(B)** Hydrophobic interactions between F112 and a patch formed by F555 and V563 are conserved in both models. This works as an anchor that allows a pivoting to the correct pose. **(C)** The movement (indicated with an arrow) is facilitated by the additional hydrophobic contacts from I79 and F81 that slide around F555.

It is thus of interest to analyze what interactions need to be present for binding to occur fast, i.e., interactions that are likely formed at the transition state for binding. The C-terminal loop of the DARPin in model r44 appears to be positioned similarly to the loop of the near-native r37 (Fig 4A). In both models, the hydrophobic interaction between F112 of the DARPin and the patch formed by F555 and V563 is conserved. This serves as an anchor that allows a smooth transition to the correct pose. Additional hydrophobic contacts are provided by I79 and F81 that may slide around F555 (Fig 4B–4C).

The described hydrophobic clamp is likely the major energetic contribution to the native funnel on the free energy landscape. Model r22, initially closer than r44 to r37 lacks this interaction and cannot diffuse to the correct orientation within the studied time frame. In fact, r22 shares some of the other native contacts, mediated by the bottom of the second repeat and the C-cap of the DARPin. This is the N123-N534 interaction, together with a second important hydrophobic contact between F89 and V533/V552. Nevertheless, unlike for r44, the energetic barrier must be too high to be crossed by sliding without complete unbinding.

We looked at the polar contacts at the interface described in Ref. [22]. Interestingly, the first hydrogen bond is formed after ~24 ns between N123-N534 (Supplementary Information S3 Fig). This then allows further polar contacts to be established. Together with the hydrophobic anchor, the contact N123-N534 forms a hinge that allows pivoting of the wrong model into the correct pose over time.

The fact that two trajectories starting from different conformations converge to the same final one is a strong evidence that the latter is a unique minimum on the free energy landscape. An important caveat, however, is that one cannot conclude from a single simulation that r44 is kinetically closer to the natively bound conformation than any other model. To verify that r44 is effectively kinetically closer to the native structure than the other models, many finite length simulations should be started from each of the model [24]. For this reason, we performed a number of simulations starting from both r22 and r44. Results show that out of 12 simulations, none starting from r22 converge to the correct bound state, while three out of 12 do when started from r44 within 50 ns (Supplementary Information, S4 Fig).

### Efb-C:C3d complex

For the Efb-C:C3d complex, the highest scoring model (r1) is a good hit, at an RMSD (Cα) of 1.6 Å from the crystal structure of the complex. However, it is followed by a crowd of false positives, i.e., structures with high Rosetta score and far from the native complex. The best hit (nearest native) is scored 63^rd^, at just 0.4 Å RMSD from the crystal structure. As the two components for docking (Efb and C3d) were derived from the complex structure and their binding surfaces thus perfectly match each other, it was expected that RosettaDock would provide a more accurate guess than for the DARPin G3:HER2_IV complex. The shape complementarity, ideal in this case, is the major factor considered in all docking functions. Nonetheless, the scores and the RMSD from the native structure correlate poorly (see S5 Fig in Supplementary Information).

**Fig 5:**
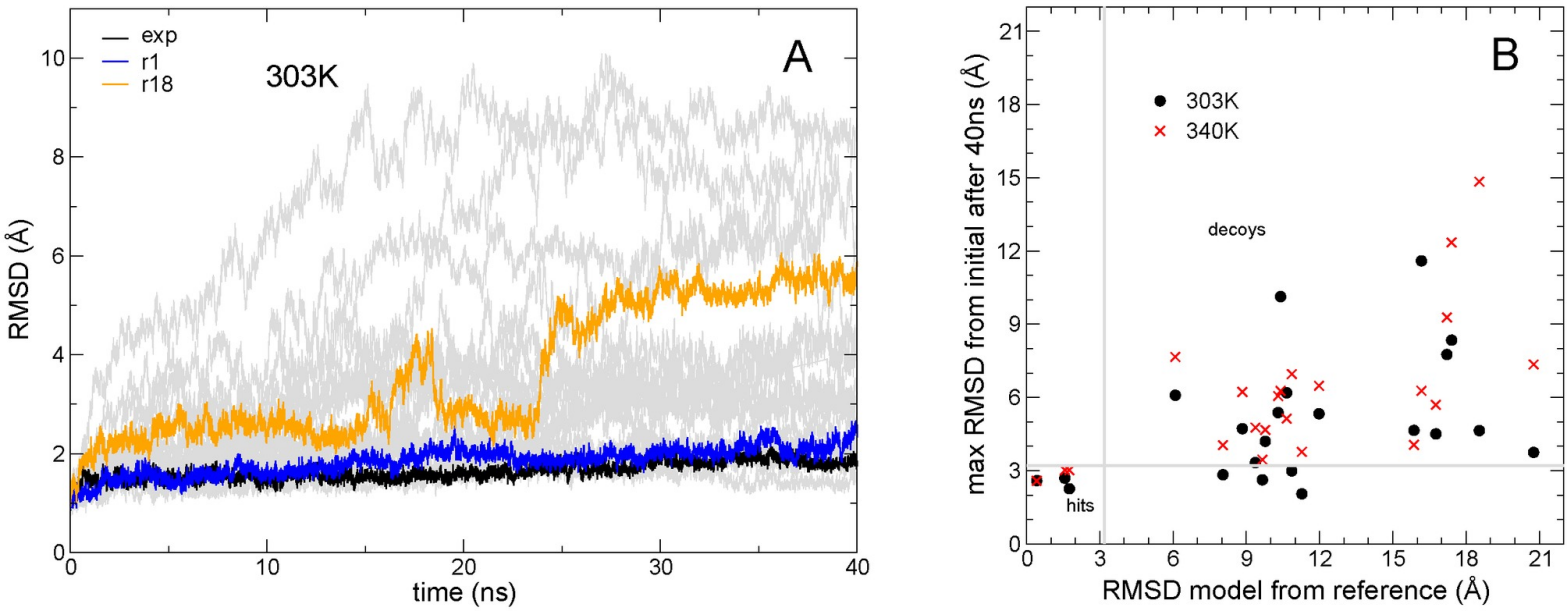
Comparison of simulations from different models of complex Efb-C:C3d. **(A)** RMSD (Cα) from the respective initial structure for the highest ranking RosettaDock models of complex Efb-C:C3d as a function of simulation time; simulations were performed at 303 K; highlighted in black is the simulation starting from the experimental structure, and in blue and orange from two selected models (also shown in Fig 6); in grey are shown all simulations starting from the 20 highest scoring models. **(B)** RMSD after 40 ns simulation plotted versus the RMSD deviation of the model from the crystal structure; simulations started from the RosettaDock models at two different temperatures; at the higher temperature (300 and 340K).

The time evolution of the structures of the various models over 40 ns simulations is shown in Fig 5A (for clarity only simulations starting from the ten top scoring models are shown). As in the previous case, the structure deviates very little from the initial one for some trajectories, and these can be identified as likely good models. Indeed, also among these models there are false positives, i.e., metastable decoys. In Fig 5B the maximum RMSD from the initial structure is shown for 22 models (the first 21 and model 63). If simulations are performed at higher temperature (340 K) (Fig 5B), the number of false positives, i.e. models that do not diverge from the initial structure and are not near-native, decreases, while the number of positives (here defined as those at less than 2 Å RMSD from the experimental structure) does not change. In other words, those that do not move away from the initial structure are the nearest native ones. The four models (Fig 5B) that during the 40 ns simulation are always within 3.2 Å from the initial structure turn out to be the ones closest to the correct structure (with an RMSD less than 2 Å from the experimental structure), while all the other models, which end up at more than 3.2 Å from the initial structure during the simulation are the ones more than 6 Å RMSD away from the experimental structure.

**Fig 6:**
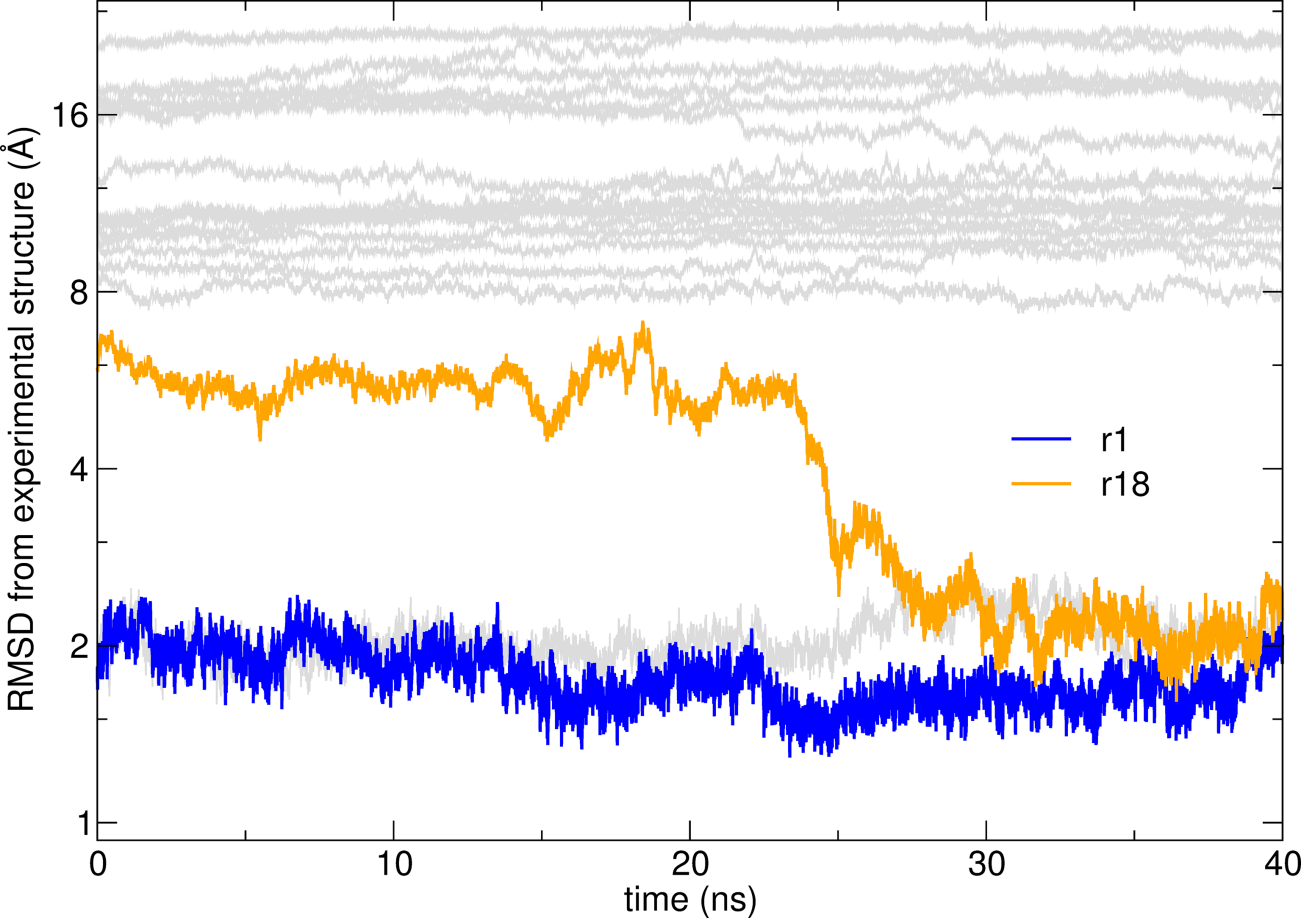
RMSD from the reference experimental structure as a function of time along simulations at 303 K for the models of Efb-C:C3d complex. Model r1 (blue) is close to the experimental structure and stays stable throughout the simulation. The trajectory starting from model r18 (orange) converges to the correct structure after about 30 ns.

As in the other case, we identified a decoy that diffused to the correct orientation. After 23 ns model r18, initially at about 7 Å RMSD from the experimental structure, starts moving towards the native conformation (Fig 6). The most conserved interacting residue between the model and the native state is R131 (Supplementary Information S6 Fig). Although its rotamers differ significantly, both are able to make a salt bridge to D1029 in the receptor. After 25 ns, Q134 and then N138 in the ligand find their right positions.

## Discussion

Discriminating near-native decoys in a crowd of false-positives is a fundamental challenge in protein-protein docking[25]. The score of a model does not generally correlate with the deviation of a model from the correct structure of the complex. This is due to a number of approximations taken by the docking algorithms to allow vast sampling of alternative conformations in reasonable time, ranging from simplified physical forces to purely statistical terms that are biased to the database used for training. Here we show that exploring the free-energy landscape around a hypothetical structure of a complex is a viable method for determining if a structure is close to a minimum of the free energy or within the funneled region of the free energy landscape where binding can occur on short timescales.

The approach we implemented here is simple: it consists in performing all-atom, fully solvated simulations of the systems in question, starting from the top-scoring models of complexes provided, in this case, by RosettaDock. Two scenarios are evident: in some simulations, the complex drifts away from the initial structure, while in others it remains close. The former could be identified as wrong models (“negatives”), and the latter as correct models of the real structure of the complex (“positives”). This was found to be often correct, but false negatives and false positives also occur. False positives result when the structure of the complex remains close to the initial structure because of a kinetic trap of the free energy landscape. False positives can be identified by performing simulations at high temperature, where metastable conformations have more chances to be overcome even in relatively short simulations. Indeed, the results presented here show that correctly docked complexes are stable even at (moderately) high temperature and most incorrectly docked complexes unbind during simulations. In all cases, this approach leads to a considerable reduction of false positives. Simulations longer that those performed here would reduce the number of false positives even further.

DARPin G3:HER2_IV is a remarkable case where essentially all the wrong models could be identified by monitoring the deviation from the initial structure by increasing the temperature up to 390 K. This may have been possible due to the very high stability of both components of the complex, where the proteins do not unfold even at very high temperatures (at least on a ~100 ns timescale). For many proteins such harsh temperature treatment may destroy their native fold.

Here we refer as false negatives to models that are kinetically unstable, but drift rapidly towards the correct structure. This demonstrates the existence of a relatively broad funnel on the free energy landscape around the correctly docked conformation.

An example here is the behavior of model r44 for G3:HER2 and model r18 for Efb-C:C3d that illustrates how a decoy may fall down the native free energy funnel and drift towards the correct structure of the complex. The models are initially at 5–8 Å RMSD from the correct structure but converge to the correct structure within 30–50 ns and remain there for the duration of the simulation.

Knowledge of the correctly docked structure was necessary here to detect such spontaneous binding events. However, even with the relatively small number of simulations, a decision on what the best model is may be made by observing the convergence of pairs of simulations to the same structure (i.e., within an RMSD that is typical of the correctly bound state for the specific complex). In Supplementary Information S1 and S2 Tables we report a list of pairs of models that at the end of the room temperature simulation are similar. For G3:HER2 the three top pairs include the two models that were correct or almost correct and the model that converges to the correct structure. For Efb-C:C3d the closest pair consists of two models that are similar to each other and at about 7 Å RMSD from the correct structure and do not drift away during the simulation; the second closest pair are models that are different (more than 7 Å RMSD from each other), and end up virtually identical after the simulation, which is a strong indication of both being within the binding funnel. Hence, in both cases a candidate for the correct model could have been uniquely identified even if the correct structure had not been available.

The results presented here highlight that empirical scoring functions are relatively good estimators of the thermodynamic stability of a protein-protein complex state, but because of relatively small energetic differences between unbound and bound states of proteins, even small errors in scoring functions may lead to false identification of the native state. A molecular dynamics simulation, where entropic and solvation effects are explicitly present, provides, albeit still approximate, an initial representation of the free energy surface over which the system diffuses. The overall shape and gradients appear to correspond to those of the real free energy surface: states with a low free energy but not confined by barriers in free energy diffuse away; correctly docked conformations appear to be kinetically stable; conformations within the funneled region of the free energy surface rapidly (in tens of ns) reach the correctly docked conformation. One take-home message is that atomistic force fields, particularly when solvent is considered explicitly, are sufficiently reliable. But most importantly, results show that the free energy landscape has been sculpted by evolution to be robust and that small errors in the atomistic force field do not alter the basic feature of there being a funnel around the biologically relevant conformation [26].

On the other hand, protocols such as RosettaDock thoroughly explore a large number of structurally diverse low energy conformers. Exploring the free energy surface by starting many independent short simulations from each of them, and possibly building a Markov state model from those [27], may become a viable way to determine the crucial features (minima and barriers) of the free energy surface and determine with high confidence the correctly bound state. The same holds true for predicting protein structures from sequences, although this is more challenging because of the much larger number of conformers that *ab initio* structure determination methods need to explore to identify models that are either correct or within the folding funnel. Therefore, all successful methods for structure prediction contain empirical terms taken from known structures to drastically reduce the search space. Similarly, empirical terms inherent in the docking programs mentioned in the Introduction are useful to decrease the initial search space, but are by themselves insufficient for the final ranking.

In conclusion, we have shown that molecular dynamics simulations starting from such models of docked complexes evolve depending on the local properties of the free energy surface. Even relatively short simulations starting from a large number of conformers selected by RosettaDock provide a valuable initial map of the free energy surface revealing the existence of more or less deep regions on the free energy surface.

## Methods

Docking was performed with RosettaDock, a part of the Rosetta 3 suite, with *talaris2014* as the scoring function during the refinement stage (details in Supplementary Methods) [28].

For the DARPin G3:HER2_IV complex, experimental structures of the unbound partners (i.e., not taken from the complex) were obtained from PDB:2jab chain A, residues 21–135 (DARPin G3) and PDB:1n8z chain C, residues 509–579 (HER2_IV). For better comparison to the known crystal structure of the complex, only the sequences corresponding to the resolved residues in PDB:4hrn (chain B and C) were considered. The structures were separately relaxed with all-heavy atom constraints, combined into one PDB file and docked via *docking_protocol*.*linuxgccrelease*. The number of trajectories was set to 10^5^ (details in the Supplementary Methods). The top 50 poses (according to total score values) were analyzed. We refer to models according to their ranks (where r1 is best and r50 is worst).

For the Efb-C:C3d complex the structure of the monomers was taken from the bound structure of the complex (PDB:2gox, chain A and B). Monomers were re-docked with Rosetta to generate 5×10^4^ poses. The top 21 models were further analyzed.

All-atom molecular dynamics simulations were performed starting from each of the highest scoring Rosetta models and from the crystal structures of the complexes. After a brief energy minimization, the models were solvated with enough water molecules so that the initial structure, when immersed in a periodic cubic box, is more that 16 Å apart from its closest image, and neutralizing ions (12 K^+^ for the DARPin G3:HER2_IV complex and 7 Cl^-^ for the Efb-C:C3d complex) were added. The CHARMM36 force field [29] was used for the proteins and the standard TIP3P model [30] for water. A timestep of 2 fs was used to integrate the equations of motion, while all bonds involving hydrogen atoms were constrained. A cutoff of 12 Å was used for the interactions and the particle mesh Ewald method was used for electrostatics. Pressure was kept constant at 1 atm using a Langevin piston, while Langevin dynamics with a low damping coefficient (1 ps^-1^) were used to keep the temperature constant. Simulations were performed using NAMD [31].

## Supporting information

S1 Fig Top 50 models and the native structure (green) of the G3:HER2_IV complex. The models are diverse and cover different surface areas of the receptor.
(TIF)

S2 Fig Key interaction surface in model r23 of complex G3:HER2_IV. The metastable binding is mediated mostly by Y46 and R23 of the DARPin.
(TIF)

S3 Fig Distance of key polar interactions in complex G3:HER2_IV as a function of time along the simulation starting from model r44. None of the interface contacts present in the crystal structure are present in r44, but after ~24 ns they begin to form.
(TIF)

S4 Fig For two of the models of the G3:HER2_IV complex 12 additional simulations have been performed by changing the initial velocity and performing a preliminary equilibration (25 ps). The purpose of the simulation was to verify the hypothesis that model r44 is kinetically closer to the native bound state than other models (e.g., r22). The additional trajectories were 40 ns long; these were restarted for another 40 ns if the RMSD from the native structure ended up being lower than the initial one after the initial 40 ns. **(A)** Trajectories started from r22 never get any closer to the native state (~5Å), while in three out of 13 cases we observe binding events when trajectories are started from r44 (colored in orange, blue and green). **(B)** RMSD from the initial structure for the same simulations started from models r22 (blue) and r44 (red). Trajectories started from r22 visit a much more limited region around the initial conformation than those started from r44. The two plots together suggest that r22, while nearer to the native conformation, is a kinetically metastable state; on the other hand, r44, which we suggest lies in the binding funnel of the free energy landscape, is an unstable state that rapidly binds with sizeable probability, and thus appears to have features that suggest it is at, or close to, the transition state for binding.
(TIF)

S5 Fig Root mean square deviation from the crystal structure of the Efb-C:C3b complex. **(A)** Top scoring 2000 models generated by RosettaDock, and **(B)** the top 50 from which simulations have been started. While the top-ranking model is very accurate (~2 Å RMSD from the experimental structure), the closest model (<1Å RMSD) ranks 63, and no correlation can be observed between RMSD and RosettaDock scoring.
(TIF)

S6 Fig Model r18 (orange) and the crystal structure of the Efb-C:C3b complex (blue). **(A)** Overview. **(B)** The only common residue for both poses is R131 in the DARPin that may interact with D1029 in the receptor. This interaction seems to be important for binding and allows a 90° pivoting of the ligand to the correct conformation during the 40 ns simulation.
(TIF)

S1 Table Analysis and comparisons of pairs of models for DARPin G3:HER2_IV complex. In the table are reported the RMSD difference (in Å) between pairs of models after a room temperature simulation (32 ns). Pairs are ordered according to the pairwise RMSD at the end of the simulation. In each case the ten pairs mutually closest are shown. The last column reports the change in RMSD between the models after the simulation (a negative number means that the structures differ less from each other after the simulation than the corresponding RosettaDock-generated starting models did). Highlighted in red are pairs of similar structures that are indistinguishable from structures explored by a simulation starting from the correct experimental structure (RMSD to the experimental structure < 1.4 Å). A large negative value in the last column is an indication of models that have moved considerably during the simulation and thus have converged during the simulation. We call this feature a signature of a native funnel on the free energy landscape. By observing convergence of trajectories toward highly similar structures the best models could be identified. Interestingly, the only “false positives” are models r22 and r48 of DARPin G3:HER2_IV, which show a low final RMSD but almost no RMSD change. We have shown in S4 Fig that r22 is a particularly stable decoy; r48 is very similar to r22 and during the simulation both trajectories do not diffuse away from the initial model, or towards the native structure.
(XLSX)

S2 Table Analysis and comparisons of pairs of models for Efb-C:C3d complex. In the table are reported the RMSD difference (in Å) between pairs of models after a room temperature simulation (40 ns). Pairs are ordered according to the pairwise RMSD at the end of the simulation. In each case the ten pairs mutually closest are shown. The last column reports the change in RMSD between the models after the simulation (a negative number means that the structures differ less from each other after the simulation than the corresponding RosettaDock-generated starting models did). Highlighted in red are pairs of similar structures that are indistinguishable from structures explored by a simulation starting from the correct experimental structure (RMSD to the experimental structure <2.5 Å). A large negative value in the last column is an indication of models that have moved considerably during the simulation and thus have converged during the simulation.
(XLSX)
