# Correction: Assessment of *ab initio* models of protein complexes by molecular dynamics

**DOI:** 10.1371/journal.pcbi.1006598

**Published:** 2018-11-09

**Authors:** The PLOS Computational Biology Staff

Supporting Information file S1 Text was not published with the manuscript. The publisher apologizes for the error. The Supporting Information file is available below.

## Supporting information

S1 Text Supplementary methods. (DOCX)
