# Supplementary figures and images for: Assessment of ab initio models of protein complexes by molecular dynamics
Source: PLoS Comput Biol. 2018 Jun 4;14(6):e1006182. doi: 10.1371/journal.pcbi.1006182 (PMC6002105; doi:10.1371/journal.pcbi.1006182)

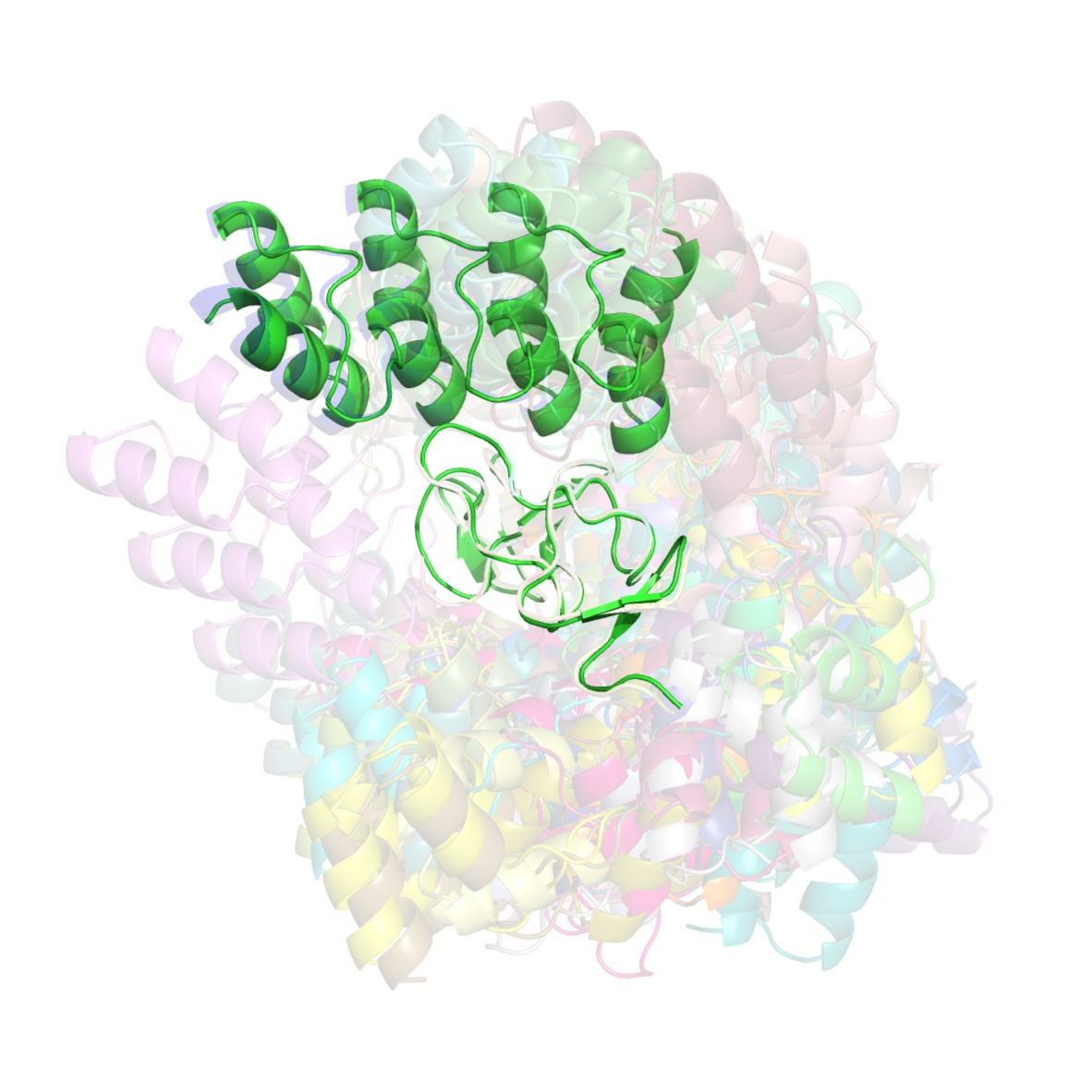

Supplement: S1 Fig — The models are diverse and cover different surface areas of the receptor. (TIF) [file pcbi.1006182.s001.tif]

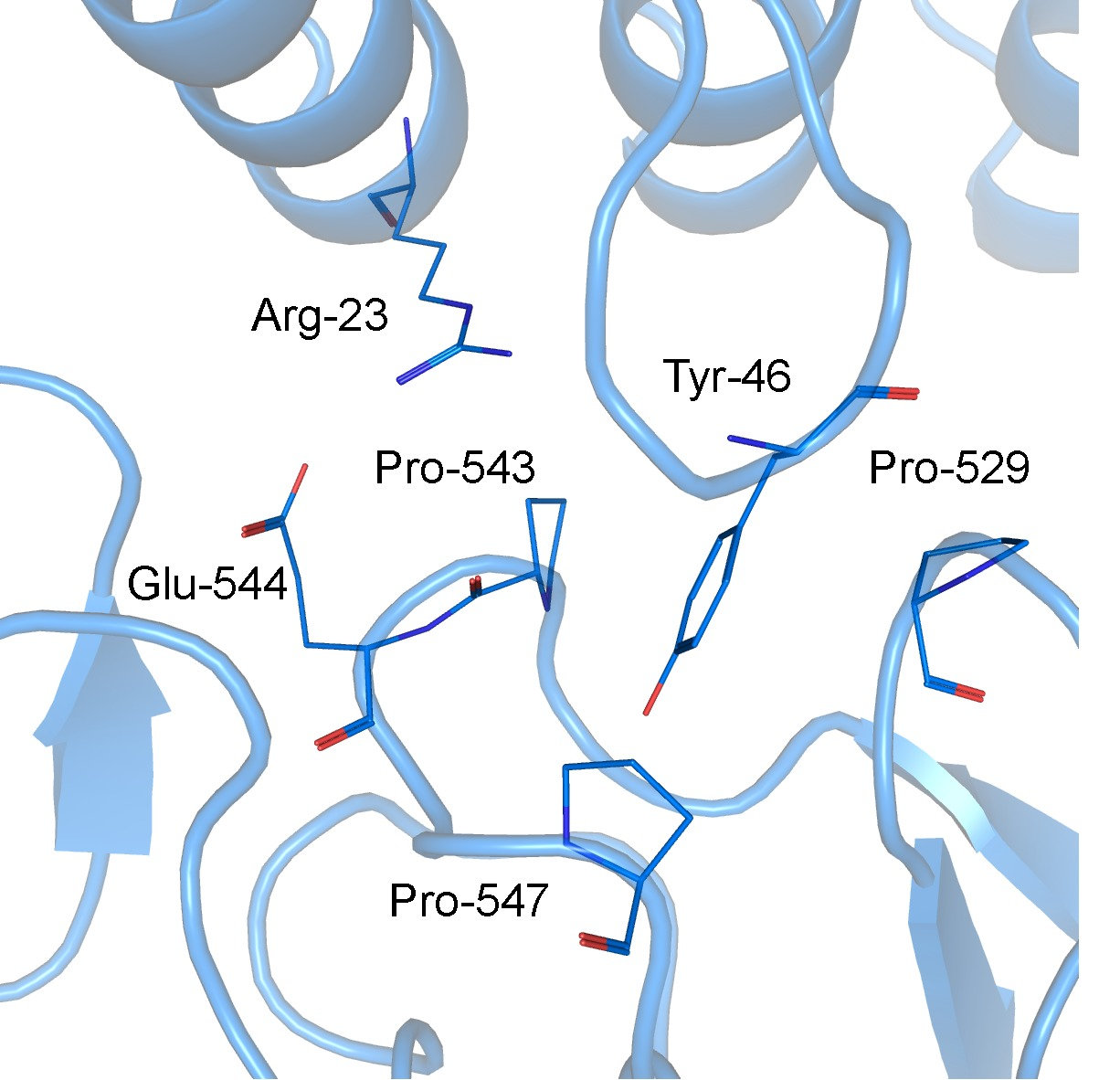

Supplement: S2 Fig — The metastable binding is mediated mostly by Y46 and R23 of the DARPin. (TIF) [file pcbi.1006182.s002.tif]

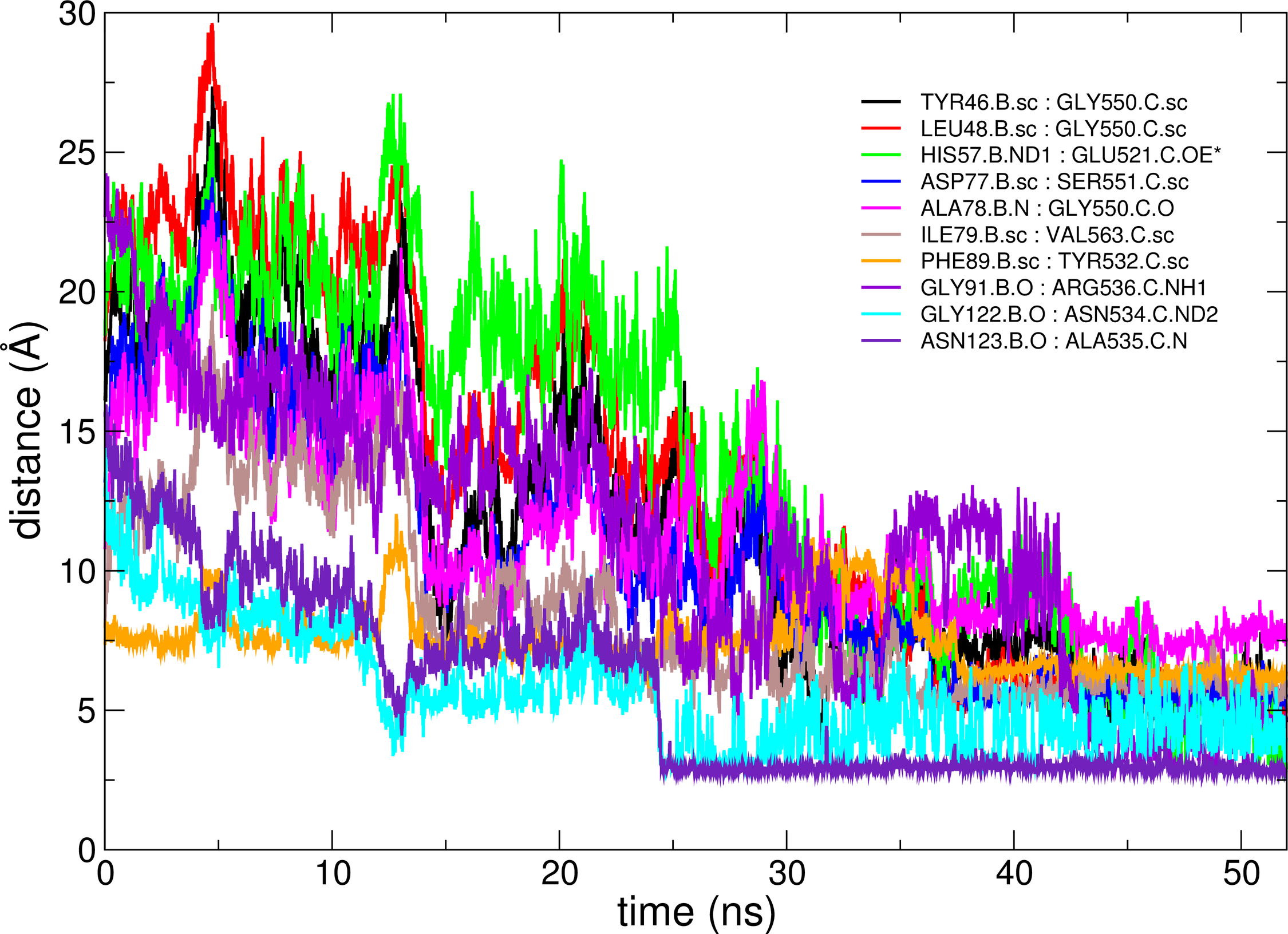

Supplement: S3 Fig — None of the interface contacts present in the crystal structure are present in r44, but after ~24 ns they begin to form. (TIF) [file pcbi.1006182.s003.tif]

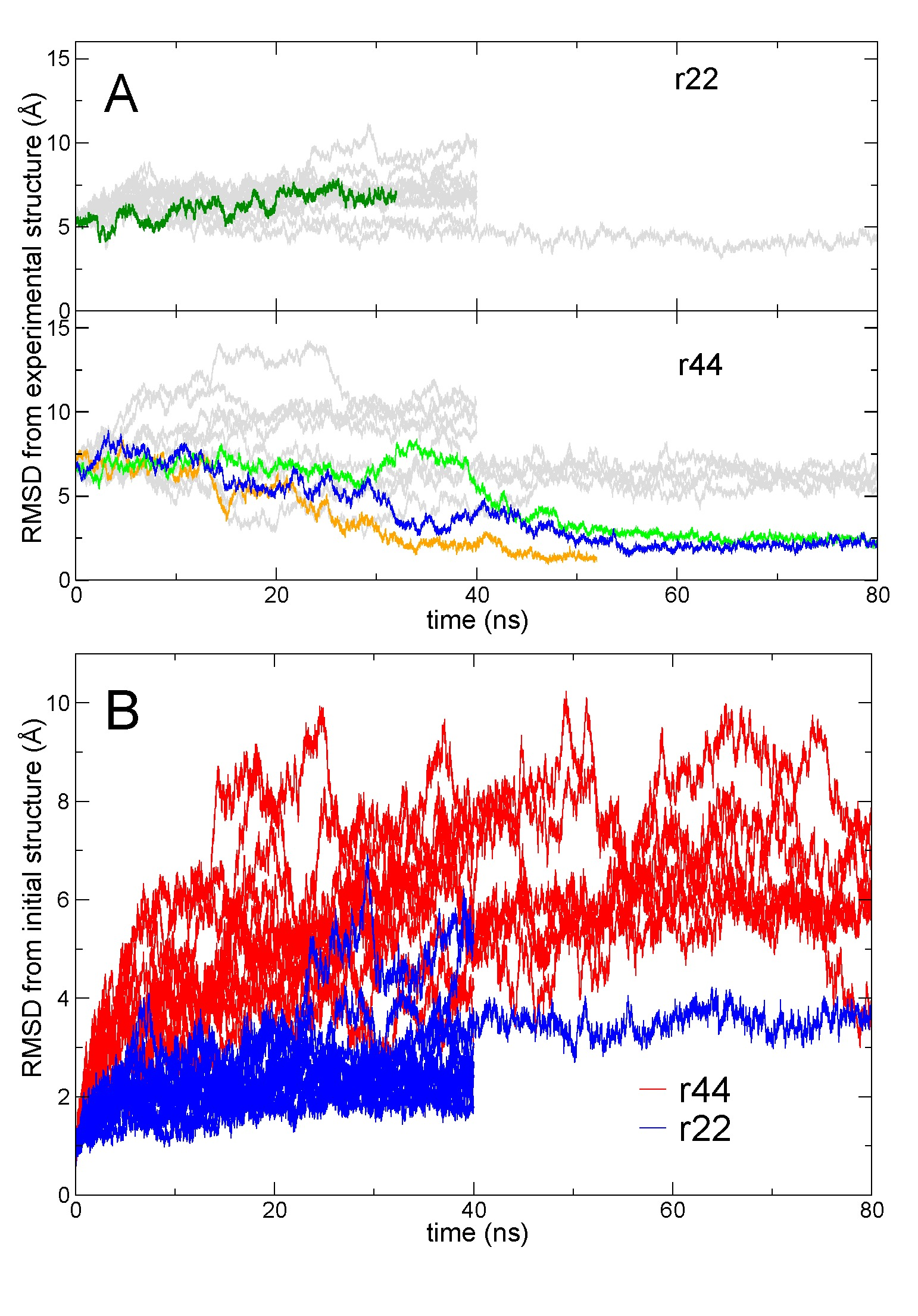

Supplement: S4 Fig — The purpose of the simulation was to verify the hypothesis that model r44 is kinetically closer to the native bound state than other models (e.g., r22). The additional trajectories were 40 ns long; these were restarted for another 40 ns if the RMSD from the native structure ended up being lower than the initial one after the initial 40 ns. (A) Trajectories started from r22 never get any closer to the native state (~5Å), while in three out of 13 cases we observe binding events when trajectories are started from r44 (colored in orange, blue and green). (B) RMSD from the initial structure for the same simulations started from models r22 (blue) and r44 (red). Trajectories started from r22 visit a much more limited region around the initial conformation than those started from r44. The two plots together suggest that r22, while nearer to the native conformation, is a kinetically metastable state; on the other hand, r44, which we suggest lies in the binding funnel of the free energy landscape, is an unstable state that rapidly binds with sizeable probability, and thus appears to have features that suggest it is at, or close to, the transition state for binding. (TIF) [file pcbi.1006182.s004.tif]

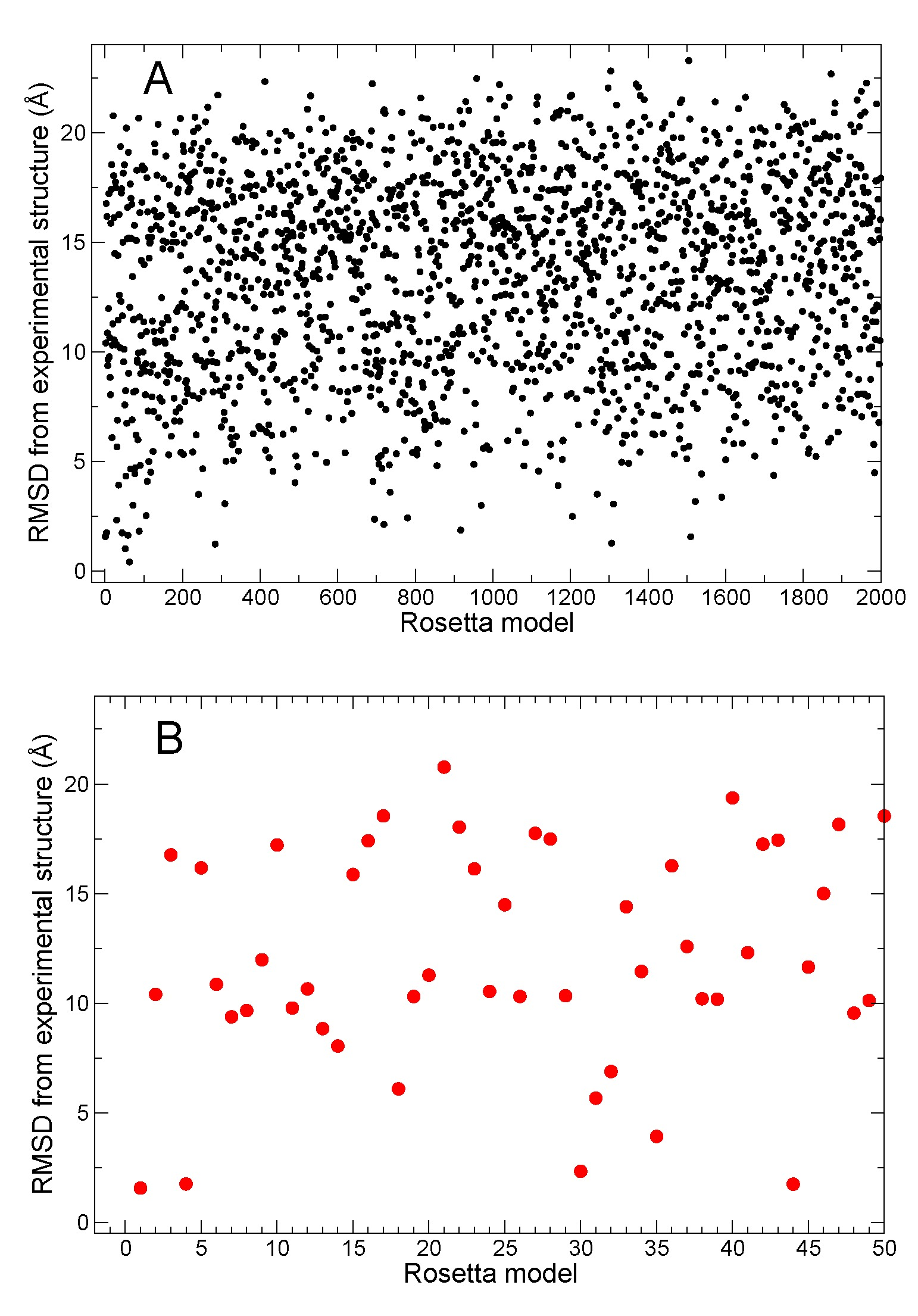

Supplement: S5 Fig — (A) Top scoring 2000 models generated by RosettaDock, and (B) the top 50 from which simulations have been started. While the top-ranking model is very accurate (~2 Å RMSD from the experimental structure), the closest model (<1Å RMSD) ranks 63, and no correlation can be observed between RMSD and RosettaDock scoring. (TIF) [file pcbi.1006182.s005.tif]

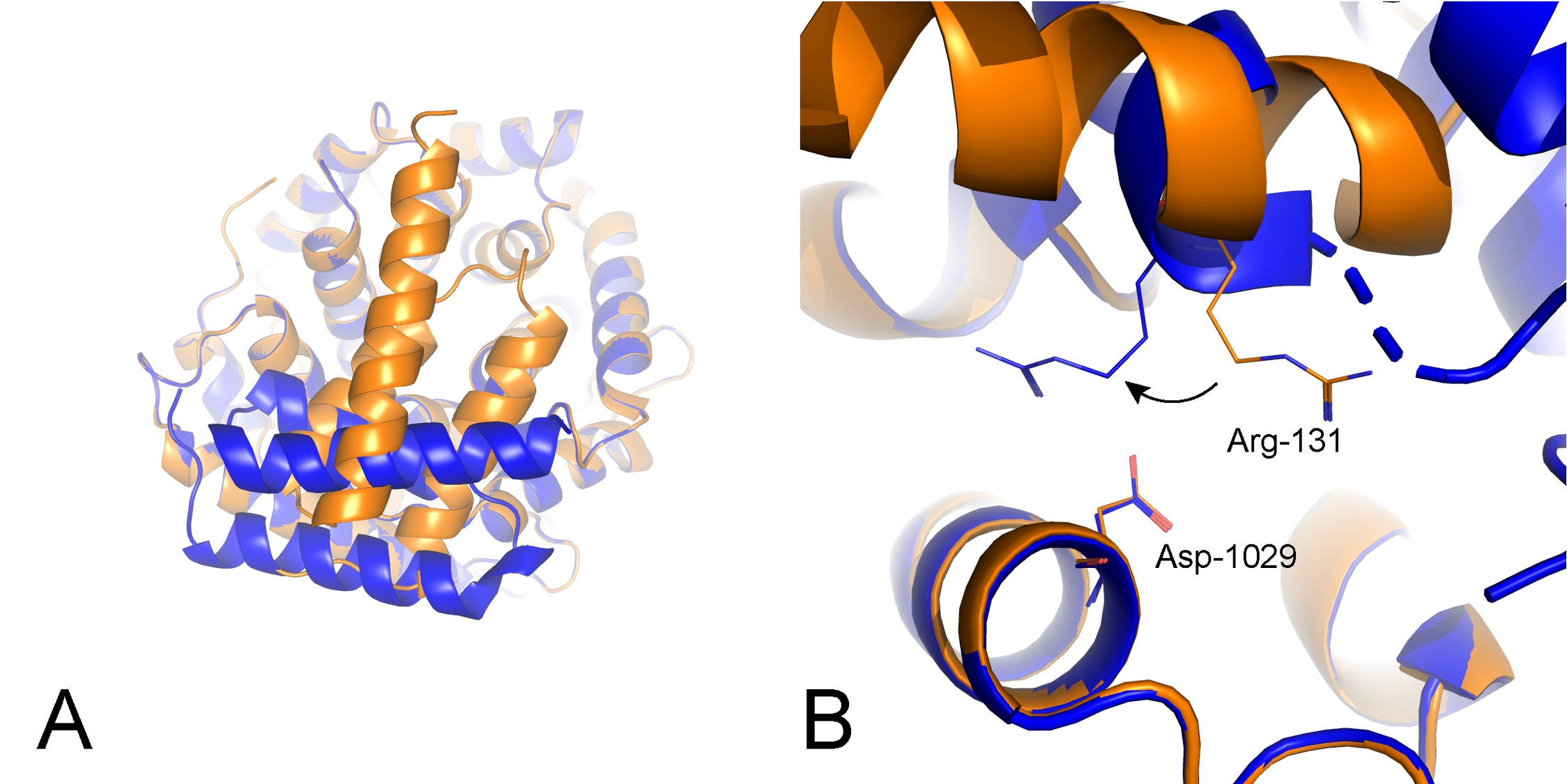

Supplement: S6 Fig — (A) Overview. (B) The only common residue for both poses is R131 in the DARPin that may interact with D1029 in the receptor. This interaction seems to be important for binding and allows a 90° pivoting of the ligand to the correct conformation during the 40 ns simulation. (TIF) [file pcbi.1006182.s006.tif]
