# Supplementary material for: Correction: Assessment of ab initio models of protein complexes by molecular dynamics
Source: PLoS Comput Biol. 2018 Nov 9;14(11):e1006598. doi: 10.1371/journal.pcbi.1006598 (PMC6226145; doi:10.1371/journal.pcbi.1006598)
Supplement: S1 Text — (DOCX) [file pcbi.1006598.s001.docx]

# Supplementary Methods

Rosetta version 58424 was used. The Rosetta command-line arguments were as follows.

## Docking of DARPin G3:HER2_IV

*relax.linuxgccrelease\*

-database ~/Rosetta/main/database\

-in:file:s ./name.clean.pdb\

-in:file:fullatom\

-out:path:all ./rosetta_output\

-out:file:scorefile name.sc\

-nstruct 1\

-ex1\

-ex2\

-use_input_sc\

-flip_HNQ\

-no_optH false\

-relax:constrain_relax_to_start_coords\

-relax:coord_constrain_sidechains\

-relax:ramp_constraints false

*docking_protocol.mpi.linuxgccrelease\*

-database ~/Rosetta/main/database\

-in:file:s ./name.clean.relaxed.complex.pdb\

-out:path:all ./rosetta_output\

-out:file:scorefile name.sc\

-out:file:silent name.out\

-nstruct 100000\

-ex1\

-ex2aro\

-use_input_sc\

-partners C_A\

-randomize1\

-randomize2\

-spin\

-score:docking_interface_score 1

## Docking of Efb-C:C3d

*docking_prepack_protocol.linuxgccrelease\*

-in:file:s ./2gox.clean.pdb\

-out:path:all ./\

-docking:partners A_B

*docking_protocol.mpi.linuxgccrelease\*

-database ~/Rosetta/main/database\

-in:file:s 2gox.clean.prepacked.pdb\

-out:path:all ./rosetta_output\

-out:file:scorefile name.sc\

-out:file:silent name.out\

-nstruct 50000\

-ex1\

-ex2aro\

-use_input_sc\

-partners A_B\

-randomize1\

-randomize2\

-spin\

-score:docking_interface_score 1

## Abbreviated protocol for detection of correctly bound conformation from an ensemble of RosettaDock models

In the main paper we presented two approaches to assess the kinetic stability of models of complexes that we summarize here as an abbreviated protocol.

1. Generate a large number (~1000) of models with the global docking method of choice. We used Rosetta, but several other algorithms are available, often in form of automated servers.
2. Pick the ~50 with highest score.
3. Start a fully solvated simulations at room temperature for ~40 ns from each model.
4. Stop simulations that diverge more than ~5 Å from the initial model (Cα RMSD) (the

threshold should depend on the system, and it should be larger for flexible proteins that display hinge movements, have flexible loops, etc.).

1. Restart simulations that ended without reaching the RMSD threshold, increasing the temperature by ~20 K.
2. Repeat step 3 and 4 until most conformers have diffused away and consider the remaining ones as candidates for the correct bound state.

Alternatively, if resources allow,

1. Perform longer (~100 ns) simulations at a single temperature (e.g., 300 K).
2. Monitor the RMSD deviation between every pair of simulations at definite time intervals.
3. Identify “convergence” between pairs of trajectories (i.e., if conformations become indistinguishably similar at the end of the simulation). Such pairs may reveal that one or both trajectories have reached a broad minimum of the free-energy. If several trajectories remain stable around a particular conformation, this is a strong clue that the correct bound state has been reached.
